# The clinical placement learning environment: a cross-sectional study of the perception of third- and fourth-year medical imaging students in Ghana

**DOI:** 10.1186/s12909-025-08207-2

**Published:** 2025-11-14

**Authors:** Wuni Abdul Razak, Akpabli Eric, Messiah Narh Kwame Anudjo

**Affiliations:** 1 Department of Radiology and Medical Imaging, Fatima College of Health Sciences, Al Ain, United Arab Emirates; 2 Department of Nuclear Safety and Security, University of Ghana, Accra, Ghana; 3 Institute of Medical Imaging and Visualisation, Faculty of Health, Environment and Medical Sciences, Bournemouth University, Bournemouth, United Kingdom; 4 School of Allied Health Professions and Pharmacy, Faculty of Medicine and Health Sciences, Keele University, Keele, Staffordshire, United Kingdom

**Keywords:** Clinical placement, Learning environment, Medical imaging students, Student perception

## Abstract

**Introduction:**

Clinical placements are critical in bridging theoretical knowledge and practical skill gaps in medical imaging education. This study evaluated the perceptions of the clinical placement learning environment among third- and fourth-year medical imaging students in Ghana.

**Methods:**

A quantitative cross-sectional survey was conducted through a self-administered questionnaire created using Google Forms. A convenience sampling strategy was employed, and the link to the Google Form was distributed via the WhatsApp platform of Medical Imaging students. The survey evaluated supervision and support, learning integration, and clinical environment equity among the participants. Descriptive and Spearman’s rank correlation (*P* < .001) were used to analyse the data.

**Results:**

A total of 253 radiography students participated, with the majority being male (65.2%) and aged 18–24 years (85.8%). Significant associations were noted in supervision and support (*p* < .001), learning integration (*p* < .001), and learning environment equity (*p* < .001). However, perceived challenges such as overcrowding (25.9%), increased workload (25.5%), and equipment breakdowns (18.9%) were significant concerns. Significant associations were observed among the measured variables (that is, supervision and support, learning and integration and learning environment equity) (*p* < .01).

**Conclusion:**

While students generally reported positive experiences, challenges such as overcrowding, limited supervision, and resource constraints hinder optimal learning. Addressing these issues through a timely and effective maintenance culture, improved infrastructure, and enhanced coordination between academic and clinical settings is crucial for fostering a supportive and equitable clinical placement environment.

## Introduction

 A key objective of higher education is to equip students with the tools and strategies needed to effectively apply the knowledge and skills acquired in one context to other learning environments [1–5]. When students fail to achieve this goal, it is viewed as a challenge, particularly considering that the seamless transition between classroom learning and clinical practice is a fundamental aspect of effective learning [6–8]. Clinical placements are vital to medical imaging education, bridging the gap between theoretical knowledge and practical applications in the real world [9, 10]. These experiences enable students to acquire the essential knowledge, skills, and professional attributes required for clinical radiography practice [11]. Comprehensive health education integrates classroom-based theoretical instruction [3], providing a scientific foundation for the profession, with clinical placements, which play an indispensable role in shaping students’ professional competence and capabilities [10, 12, 13]. Typically, students in medical imaging programs spend most of their training in clinical placements, where they observe professionals, interact with patients, and perform procedures under supervision [14], which enables them to build key professional competencies.

During clinical placements, consistent monitoring is essential for students. According to Butterworth and Faugier [15], clinical supervision serves as a structured support system for healthcare professionals aimed at fostering competence and professionalism. Clinical supervision involves an experienced healthcare professional guiding the clinical practice and professional development of a less skilled individual [16]. At the undergraduate level, the emphasis is primarily on clinical teaching and assessment, and effective clinical supervision enhances the quality and safety of patients while fostering a supportive learning environment [17, 18]. 

The integration of theoretical and practical knowledge is crucial for effective clinical training and the development of competent medical imaging professionals [19]. The structure and implementation of radiography education emphasise the seamless integration of classroom instruction with clinical training, as effective patient management is both a fundamental responsibility and an essential component of healthcare education [20, 21]. However, this has not consistently been the case, as a significant knowledge gap persists in connecting classroom learning to clinical placement, as shown by Karen and colleagues (2023) [22]. These gaps may stem from factors such as unclear role definitions, time constraints, heavy workloads, and insufficient resources [21, 23, 24]. 

An integrative review of global studies conducted by Jessee [25]on CLE revealed that supervisory relationships, peer relationships and the clinical education structure are among the key factors consistently affecting students’ perception. Additionally, a comparative study of 558 nursing students in Finland (*n* = 416) and the UK (*n* = 142) revealed that Finnish students reported a more positive perception of their CLE, with greater satisfaction regarding the overall environment and nurse-teacher interactions [26]. Also, a national survey conducted in Thailand among 2,467 medical students indicated an overall positive perception of the CLE [27]. A qualitative study conducted in Rwanda, a resource limited setting, among 25 medical imaging students found that students faced significant challenges due to the theory-practice gap, insufficient teaching support and resource limitations [14]. While the core factors influencing CLE perceptions are consistent across regions, contextual factors such as availability of resources and the structure of clinical education can significantly impact students’ CLE.

In Ghanaian radiography education, students typically commence clinical practice in their second year of study. Clinical placements are organized and coordinated by academic institutions. Students are placed in clinical settings across a range of healthcare facilities with assignments determined by educational institutions in collaboration with the placement site. Academic institutions coordinate the placement of students, ensuring that each student is assigned to a clinical site that aligns with their training needs. Clinical placements site is earmarked based on the number of modalities the facility has, number of facilities willing to train students, and the proximity of the clinical site to the students’ primary residence. Students are usually placed in groups, with each group having at least 5–10 students assigned to a clinical radiographer (practitioner). Students usually rotate within the Imaging department depending on the number of modalities the clinical placement has and spend at least 3 months having hands on training these sites. At the end of the students’ clinical placement, they are assessed and graded by the clinical radiographer which contributes to the overall grading of the students’ assessment.

This phase is designed to help students bridge the gap between the theoretical knowledge acquired in the classroom and its practical application in medical imaging [28, 29]. Supervisors from academic institutions visit students in their clinical learning environments to assess and grade their performance as a test of their learning progress. However, these supervisory visits alone may not provide a comprehensive evaluation of the effectiveness of clinical placements where they are supervised by practitioners. This study evaluated the perceptions of the clinical placement learning environment among third- and fourth-year medical imaging students in Ghana.

## Methods

### Study design and setting

A quantitative cross-sectional survey was used to evaluate the perceptions of the clinical placement learning environment among medical imaging students [30]. This quantitative cross-sectional design was selected because it allowed for the evaluation of perception and perceived challenges faced by third- and fourth-year students, providing a comprehensive overview of their viewpoints at a specific point in time. The study was conducted at 5 public institutions in the country. The institutions offering medical imaging programmes in the country include the University of Ghana (UG), University of Cape Coast (UCC), University for Development Studies (UDS), University of Health and Allied Sciences (UHAS), College of Health Kintampo, and Kwame Nkrumah University of Science and Technology (KNUST). These institutions were selected because they are the leading public universities offering well-established radiography programs and have the largest student populations in the field. Focusing on these universities allowed for a more consistent comparison across well-established educational programs.

### Study population

The study focused on third- and fourth-year undergraduate medical imaging students at Ghana’s public universities. Third- and fourth-year undergraduate degrees are considered the clinical years where clinical placement is embedded intensively; therefore, students at these levels can provide valid opinions on their clinical environment.

Students in their third and fourth years of the undergraduate medical imaging programme were eligible. Participation was limited to those who provided informed consent after being briefed on the study’s purpose and procedures. Students without clinical placement experience during the academic year of the study were excluded.

### Sample size and sampling technique

A sample size of 252 was estimated using Cochran’s formula for a finite population [31]. The sample size estimation was based off the total population (N) of third- and fourth-year students which was found to be 731. This figure was obtained from the sum of the total population of Year 3 and Year 4 students from the various selected institutions’ registers. Convenience sampling technique was used because of its practicality and efficiency in accessing participants who are readily available and willing to take part in the study [32]. This method was particularly suitable for the study, as it targeted medical imaging students at specific universities, allowing for quick data collection from this accessible population with the requisite characteristics ideal for addressing the research question.

### Data collection instrument and procedure

A structured, self-administered questionnaire was created using Google Forms, and the link to the form was shared on the WhatsApp platform of Medical Imaging students. The research instrument used was a validated questionnaire divided into two parts. Part one consisted of closed-ended questions on students’ demographics, gender, age, clinical year, and institution. Part 2 was the 25-item Undergraduate Clinical Education Environment Measure (UCEEM) developed by Strand [33]. Part two was scored on a five-point Likert scale (ranging from 1 = fully disagree to 5 = agree to a large extent) and a categorical scoring system based on the percentage distribution of responses for each item was used. To classify perceptions as positive, the combined percentage of responses in the agree to a large extent and fully agree categories was greater than 50% and negative perceptions had the combined percentage of responses in the agree to a slight extent and fully disagree categories was less than 50%. UCEEM covers two broad areas: experiential learning and social participation. It also had the following subareas: (1) Opportunities for learning through work and the quality of supervision, (2) how prepared the facility is for students’ entry, (3) Social inclusion and workplace interactions; and (4) Equality of treatment. In addition to the UCEEM items, the questionnaire also included a section on challenges encountered during clinical placement. These challenges were presented as pre-listed options based on prior literature [34, 35]. and students could select all that applied.

### Validity and reliability

A pilot test was conducted among seven Year 3 medical imaging students to confirm clarity of wording and ease of completion. Minor refinements were made to improve readability and ensure alignment with local terminology based on their feedback. The final questionnaire was administered electronically via Google Forms to facilitate access, preserve anonymity, and enable efficient data collection across multiple institutions.

Content validity was ensured through expert review by members of the research team who are academics with years of experience in radiography education and health research. These experts examined the questionnaire for relevance, clarity, and alignment with the study objectives. The core items were adapted from the Undergraduate Clinical Education Environment Measure (UCEEM), an instrument that has been widely applied in health professions education and shown to capture students’ perceptions of the clinical learning environment effectively. Additional items were incorporated to reflect challenges common in Ghanaian clinical training.

Internal consistency statistics such as Cronbach’s alpha were not computed, as the questionnaire combined UCEEM items with context-specific items designed to capture multiple dimensions of the clinical placement environment rather than a single construct. Reliability was therefore assessed in terms of response clarity, consistency across pilot data, and expert review of item appropriateness.

### Statistical analysis

The data collected were analysed via the Statistical Package for the Social Sciences (SPSS) version 26 and Microsoft Excel 2019. Descriptive statistics were employed to summarise the sample characteristics and key variables: supervision and support, learning and integration, environment and equity, providing a comprehensive overview of the demographic distribution. A normality test was conducted between the variables: supervision and support, learning and integration, environment and equity, to assess the distribution of the data. Because the variables deviated from a normal distribution, nonparametric analysis using Spearman’s rank correlation was used [36]. was employed to examine the association among the variables. Statistical associations were considered significant at *p* <.05. Multiple linear regression model was also conducted to determine the influence the independent variables (Supervision and Support, and Environment and Equity) have on the dependent variable (Learning and Integration).

### Ethical consideration

The study was conducted in full compliance with ethical standards, receiving ethical approval from the Education and Research Committee of the Ghana Society of Radiographers (GSR) with registration number GSR/RS/PS/FTA/002/2024. This study was conducted in accordance with the ethical principles outlined in the 1964 Declaration of Helsinki and its subsequent amendments. Participants were provided with a detailed informed consent form that explained the study’s purpose, their role, and their rights, emphasizing the voluntary nature of their participation and the confidentiality of their responses. Informed consent was obtained electronically before participants accessing the survey. To protect participants’ privacy, all data was anonymized and securely stored in password-protected databases accessible only to the research team.

The research and education committee thoroughly reviewed the study to ensure compliance with institutional policies and ethical standards, addressing and resolving any ethical concerns raised. The study was examined to make sure it complied with institutional policies and procedures and was ethical. The committee resolved any ethical issues that were raised.

## Results

### Demographic data of the participants

The demographic data revealed that the majority were males (65.2%), whereas females accounted for 34.4%, with one individual preferring not to disclose their gender. Most participants (85.8%) were aged 18–24 years, 59.3% were in Year 4, and 40.7% were in Year 3. The University of Cape Coast had the highest percentage of participants (35.6%), and the College of Health Kintampo had the lowest percentage of participants (4.0%). Table 1 provides further details.

**Table 1:** Demographic data of the participants

Demographics	*N* (%)
Gender	
Male	165 (65.2)
Female	87 (34.4)
Prefer Not to Say	1 (0.4)
Total	253 (100)
Age (Years)	
18–24	217 (85.8)
25–30	33 (13.0)
31–35	3 (1.2)
Total	253 (100)
Clinical Year	
Year 3	103 (40.7)
Year 4	150 (59.3)
Total	253 (100)
Institution	
University of Ghana	19 (7.5)
University of Cape Coast	90 (35.6)
University of Health and Allied Sciences	64 (25.3)
University for Development Studies	33 (13.0)
College of Health Kintampo	10 (4.0)
Kwame Nkrumah University of Science and Technology	37 (14.6)
Total	253 (100)

### Perceptions on the clinical placement environment

The results on perceptions of students regarding clinical placement environment were analysed according to supervision and support, learning and integration, environment and equity. They were described as follows:

### Supervision and support

**Fig. 1:**
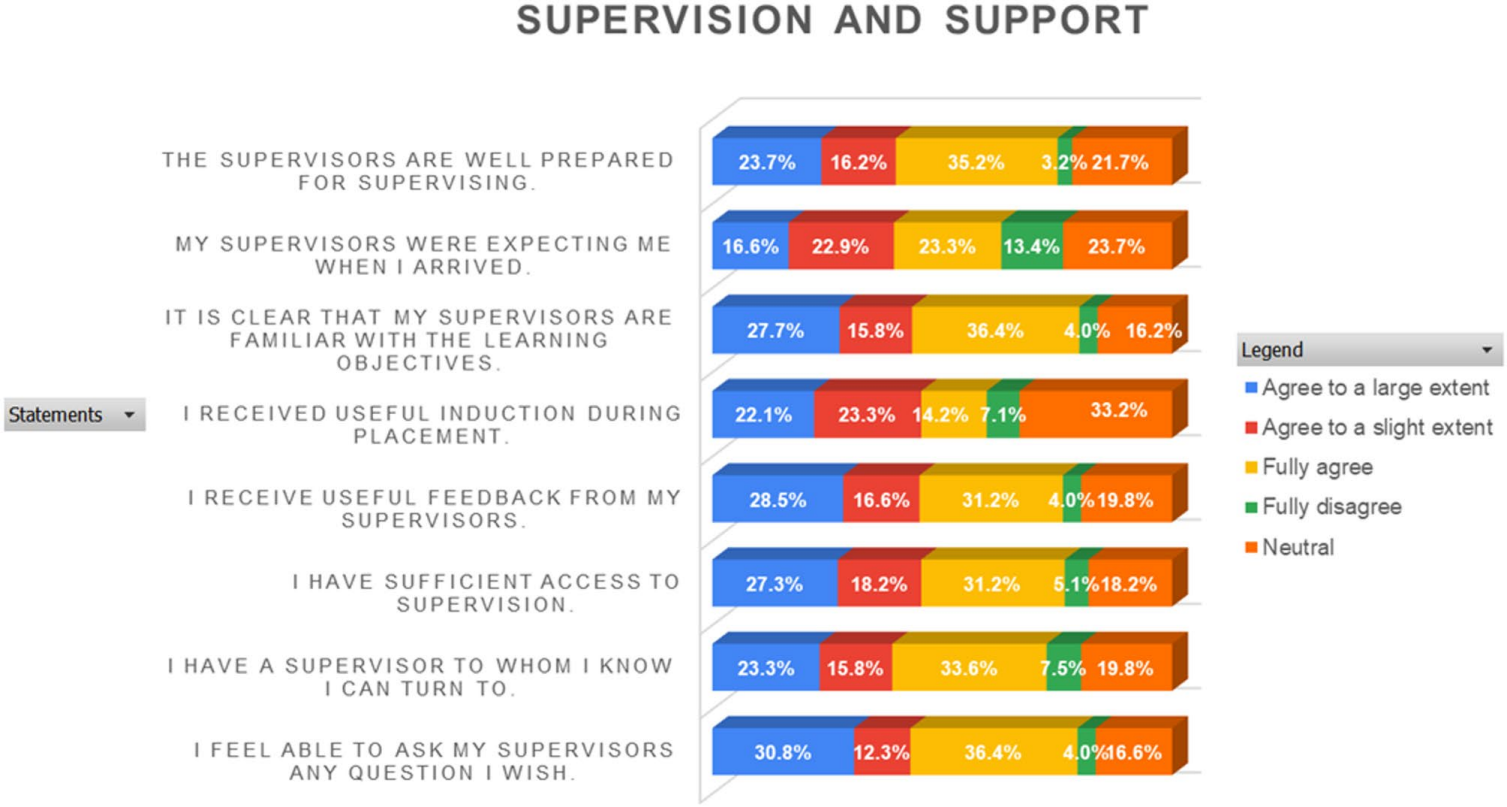
Stack bar chart analysis of likert scale questions on supervision and support (SS)

The analysis reveals a strong positive perception of supervision and support, with most respondents consistently rating these aspects as “agree to a large extent” or “fully agree.” A smaller proportion rated the support as “neutral,” whereas minimal responses fell into the “agree to a slight extent” or “fully disagree” categories, as shown in Fig. 1.

### Learning and integration

**Fig. 2:**
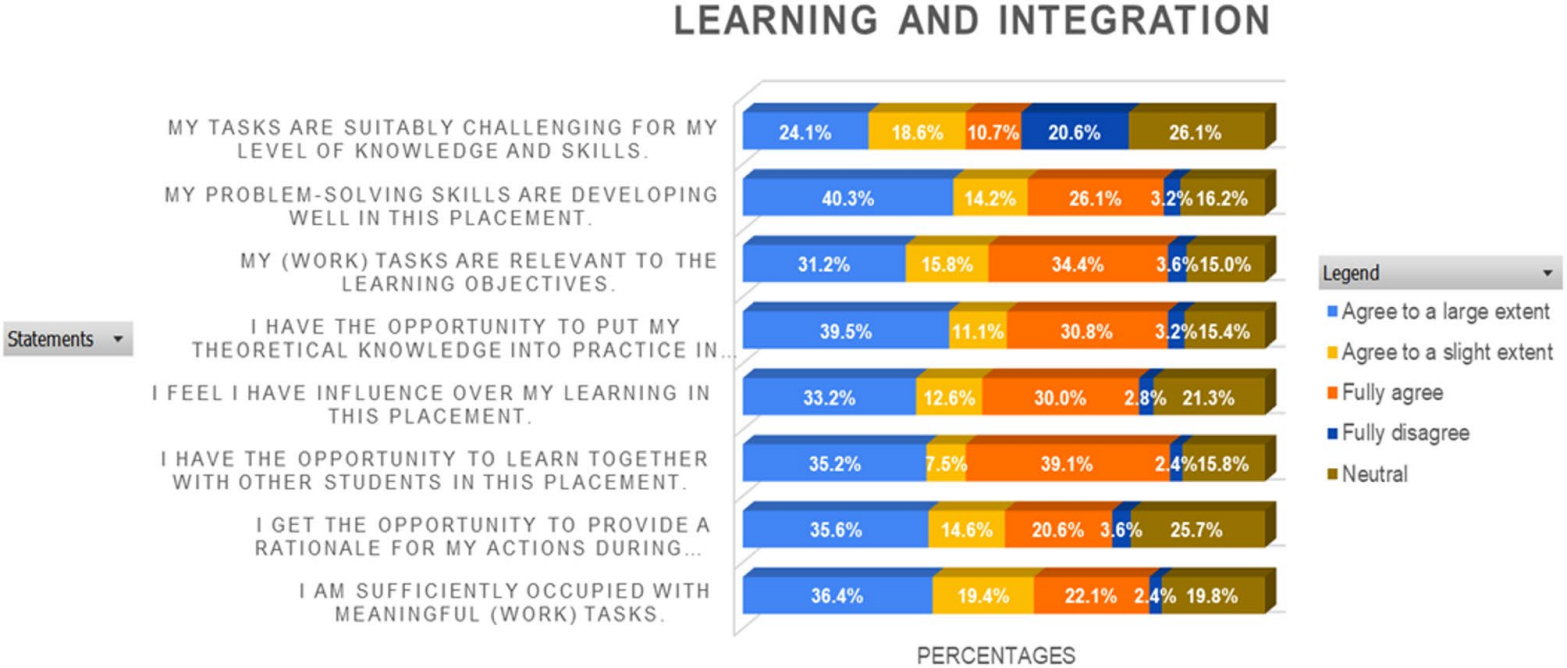
Stack bar chart of likert scale questions on learning and integration (LI)

The stacked bar chart in Fig. 2 evaluates students’ perceptions of learning and integration during their clinical training based on Likert scale responses. Most respondents rated the learning and integration experience positively, with approximately 70% selecting “Agree to a large extent” or “Fully Agree.” Approximately 25% expressed a “neutral” stance, whereas less than 5% rated the experience as “agree to a slight extent” or “fully disagree.”

### Environment and equity

**Fig. 3:**
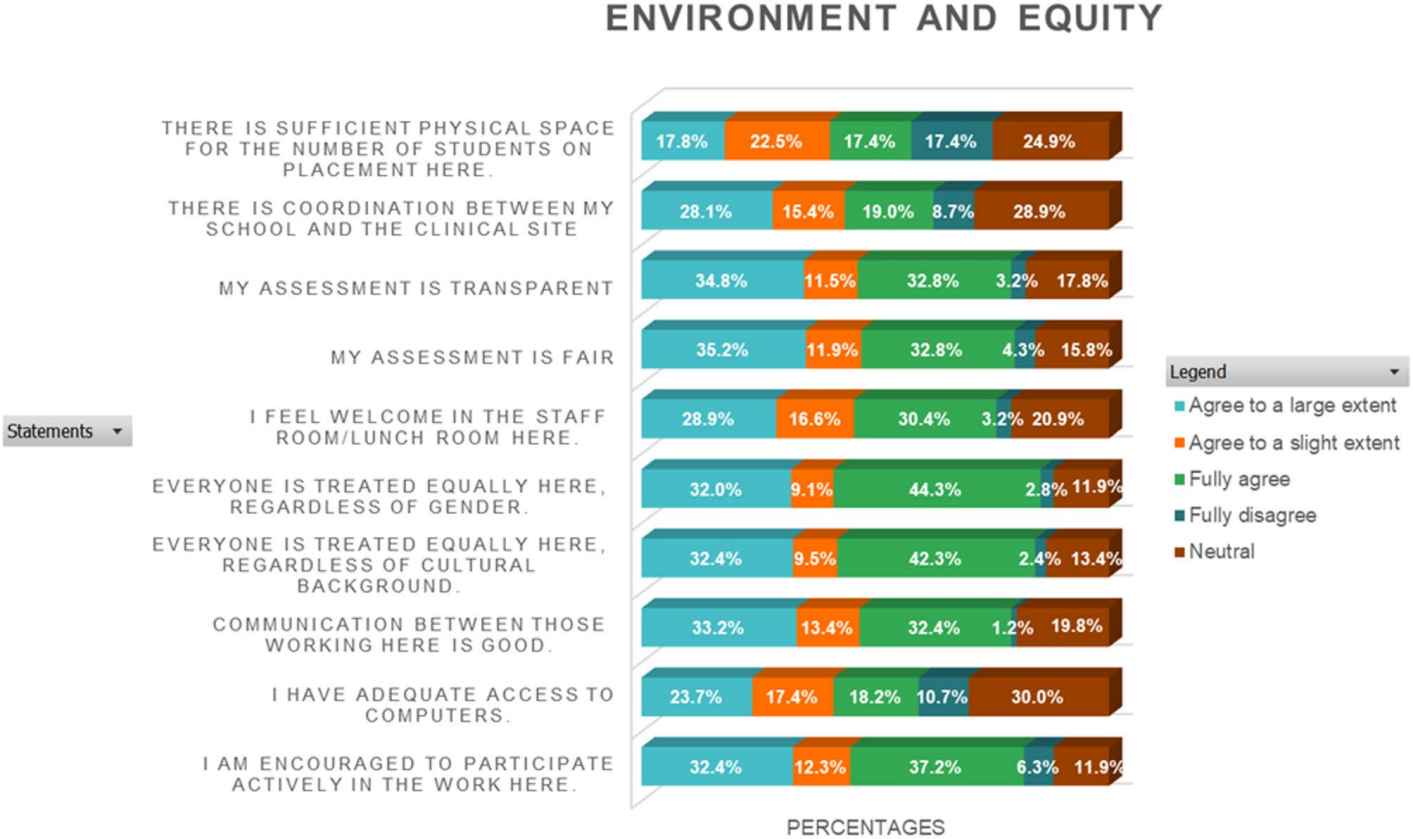
Stack bar chart analysis of likert scale questions on environment and equity (EE) in the clinical area

Most students rated the environment and equity positively, with a substantial portion selecting “Agree to a slight extent” or “Agree to a large extent.” indicating satisfaction with the inclusivity and fairness within their clinical settings. A moderate percentage provided “neutral” ratings, reflecting an ambivalent stance, whereas only a small minority rated these aspects as “fully disagree.” Further details are provided in Fig. 3.

### Normality test

The normality test results for the measured variables, i.e., supervision and support (SS), learning and integration (LI), and environment and equity (EE), via the Kolmogorov‒Smirnov test revealed that all three variables significantly deviated from a normal distribution (*p* <.05 for each variable) (Table 2). Specifically, the Kolmogorov‒Smirnov statistics are 0.077 for SS, 0.078 for LI, and 0.080 for EE, with significance values all below 0.001. These results are corroborated by the Shapiro‒Wilk test, which also indicates significant deviations from normality for all variables (*p* =.000).

**Table 2:** Normality test between the measured variables (SS, LI, EE)

	Kolmogorov-Smirnov^a^			Shapiro‒Wilk		
	Statistic	df	Sig.	Statistic	df	Sig.
SS	0.077	253	0.001	0.969	253	0.000
LI	0.078	253	0.001	0.977	253	0.000
EE	0.080	253	0.000	0.974	253	0.000

^a^Lilliefors Significance Correction

### Association between SS, LI, and EE

Table 3 presents the Spearman’s rank correlation coefficients between the measured variables; supervision and support (SS), learning and integration (LI), and environment and equity (EE). The results indicate strong, positive correlations among all three variables. For correlation between SS and LI (*r* =.811, *p* =.001), a strong positive correlation was noted; also, a strong positive correlation was observed between SS and EE (*r* =.771, *p* =.001). Similarly, LI and EE (*r* =.808, *p* =.000) saw a strong positive correlation.

**Table 3:** Spearman’s rank correlation between the measured variables (SS, LI, EE)

			SS	LI	EE
Spearman’s rho	SS	Correlation Coefficient	1.000	0.811^**^	0.771^**^
		Sig. (2-tailed)	.	0.000	0.000
		N	253	253	253
	LI	Correlation Coefficient		1.000	0.808^**^
		Sig. (2-tailed)		.	0.000
		N	253	253	253
	EE	Correlation Coefficient		0.808^**^	1.000
		Sig. (2-tailed)		0.000	.
		N	253	253	253

**. The correlation is significant at the 0.01 level (2-tailed)

### Regression model to predict the influence of SS and EE on LI

Table 5 shows a multiple linear regression analysis model used to predict the influence of SS and EE on LI. The model indicates both SS (B = 0.385; SE = 0.041; *P* =.000) and EE (B = 0.438; SE = 0.047; *P* =.000) are positive predictors of LI. Collinearity statistics are also acceptable (VIF) = 2.376. The model explains that 74.4% of the variance of LI is accounted for by SS and EE.

**Table 4:** Multiple linear regression results showing the influence of SS and EE on LI

						t		*P*-Vale		95.0% CI				Collinearity					
B		SE		β						Lower		Upper		Tolerance		VIF			
(Constant)		0.665		0.116				5.748		0.000		0.437		0.893					
SS		0.385		0.041		0.464		9.417		0.000		0.304		0.466		0.421		2.376	
EE		0.438		0.047		0.455		9.235		0.000		0.344		0.531		0.421		2.376	

Standardized regression coefficient = β, Unstandardized regression coefficient = B, Standard Error = SE, Dependent variable: L

Fit Indices: R= .846; R2 = .744; Adjusted R2=.742

### Perceived challenges

The most frequently reported issues were overcrowding (49.8%) and increased workload or stress (49.0%) (Table 5). Constant equipment breakdowns were also a notable challenge, reported by 36.4% of the students. Issues such as low numbers of supervisors (10.5%) and a lack of motivation from supervisors (34.4%) point to supervision-related challenges. Some less frequently mentioned issues include poor communication between schools and clinical sites, long distances to clinical facilities, and unprofessional attitudes from sonographers, which were represented by “Others” were reported by 2.4% of the students.

**Table 5:** Shows the frequency distribution of some common challenges faced by students during clinical placement

Challenges	*N* (%)
Constant equipment breakdown	92 (36.4)
Low number of supervisors	51 (21.2)
Overcrowding	126 (49.8)
Increase workload/stress	124 (49.0)
lack of motivation from supervisors	87 (34.4)
Others	6 (2.4)

## Discussion

The findings from this study provide valuable insights into the perceptions of medical imaging students regarding their clinical placement learning environments in Ghana. The results highlighted key findings related to supervision and support, learning integration, and the overall environment and equity experienced by students. The study achieved its minimum sample size of 252, with the majority (65.2%) of respondents being males.

The majority (85.8%) of the participants were aged 18–24 years, followed by 13.0% aged 25–30 years. The male dominance reported in this study is consistent with related literature [37, 38]. and the relatively smaller proportion of females than males in science-based careers, such as radiography, in Ghana [39, 40]. The age distribution revealed that the majority of the participants were between 18 and 24 years of age, which aligns with the findings of Van et al., [41] who argued that students typically graduate before age 25. The University of Cape Coast (UCC) had the highest response rate, possibly influenced by UCC’s larger student population.

### Supervision and support

The findings revealed that students generally perceive supervision and support during clinical placements as adequate, with the majority rating their experiences positively. This emphasised the critical role of adequate clinical supervision in fostering competence and professionalism among students, as highlighted in the literature [10, 15]. Students value supervisors who provide guidance, constructive feedback, and consistent support, which are essential for bridging classroom learning with practical application. The result of a Spearman’s correlation between SS and LI (*r* =.811, *p* =.001) as well as SS and EE (*r* =.771, *p* =.001) showed a strong positive correlation. This indicates that as supervision and support improve, students’ ability to integrate learning and engage academically also increases significantly. The finding corroborates this evidence that 58.5% of students agreed to having sufficient access to supervision.

To address disparities and enhance student outcomes, investing in supervisor training and addressing systemic challenges, such as overcrowding, are essential steps toward a more effective and equitable clinical education framework.

### Environment and equity

The clinical environment and equity received generally positive perceptions among the students, with the majority rating their experiences favourably. This is consistent with the literature that emphasises the importance of fostering inclusivity and fairness within clinical settings to enhance students’ professional growth [16, 42]. However, the moderate percentage of neutral responses highlights the variability in students’ experiences across clinical sites, suggesting that some may feel marginalised or unsupported. This echo concerns previous studies, such as those by Kyei et al. [43] Salifu et al. [44] identified resource limitations, overcrowding, and insufficient supervisor training as systemic challenges that hinder equitable learning environments.

The strong positive correlations between supervision and support, learning integration, and the environment and equity highlight the interconnected nature of these factors. Improvements in one area are likely to benefit others, reinforcing the need for a holistic approach to enhancing student experiences during clinical placements. This finding is supported by research indicating that a supportive learning environment positively influences student satisfaction and retention [45]. 

### Learning and integration

Most participants appeared satisfied with the alignment between theoretical knowledge and practical experience. This is an encouraging outcome, as the integration of classroom learning with clinical practice is crucial for developing competent healthcare professionals. Clinical preceptors must continually work to optimise teaching opportunities and address the diverse learning needs of students while ensuring the delivery of safe, high-quality, patient-centered care in a challenging clinical environment [46]. Research consistently shows that when students perceive a strong connection between theory and practice, they are more likely to engage with their education and feel prepared for real-world challenges [47–50]. However, the significant percentage of neutral responses highlights existing gaps in the integration process, indicating areas for improvement.

The predictive model conducted in Table 5 revealed that both SS and EE exert a significant positive influence on LI among medical imaging students in Ghana. The analysis shows a 74.4% predictive rate (R^2^ = 0.744; Adjusted R^2^ = 0.742). This suggests that enhanced supervision, combined with inclusivity and fairness, promotes greater engagement and integration. This aligns with prior literature [26, 51–53] emphasizing the role of structural support and an equitable environment in mitigating the theory-practice gap.

### Perceived challenges

The study revealed key challenges, including overcrowding, limited supervision, and a lack of motivation among supervisors, highlighting significant concerns related to capacity and student well-being. These challenges can be attributed to the increasing number of students in radiography training institutions, while clinical placement training sites remain the same, a common feature in low-resource settings. Overcrowding significantly impacts supervision quality because an increased student-to-supervisor ratio often exceeds manageable levels, thereby reducing student-supervisor contact times. Overcrowding due to limited placement could also reduce opportunities for sufficient hands-on practice [54, 55]. This concern aligns with previous studies suggesting that inadequate supervisory practices can lead to feelings of inadequacy and hinder students’ professional development [45, 56]. 

Furthermore, 34.4% of the respondents highlighted a lack of motivation among supervisors, indicating the need for formalised training programs to equip supervisors with mentoring skills and strategies for maintaining engagement. It is also essential for supervisors to be motivated by academic institutions to continue providing this critical service. Some of the ways could be the facilitation of free, continued professional development programmes for designated staff at clinical placement sites.

Poor coordination and communication between academic institutions and clinical sites also result in unclear expectations, leaving supervisors unaware of students’ specific learning goals and hindering the ability of supervisors to provide targeted and meaningful support. Additionally, the presence of neutral responses in the data suggests variability in experiences across different clinical settings, emphasising the need for standardised supervisory practices. This finding supports findings in the literature, indicating the need to implement structured feedback mechanisms that could further enhance the learning experience, as timely and constructive feedback remains a critical yet underdeveloped component in many clinical placements [42, 57]. 

Also, challenges associated with theory-practice integration were found to be consistent with findings from the literature, which highlights a pervasive gap in healthcare education. The students in this study reported issues such as insufficient time for practical application, overcrowding, and inadequate infrastructure, all of which hinder their ability to integrate theoretical knowledge effectively into clinical practice. These concerns align with findings by Salifu et al. [44] Manson et al. [58] and Botwe et al. [19] who identified resource limitations, poorly equipped skills laboratories, and heavy workloads as significant barriers to practical skills acquisition.

### Limitations of the study

Notably, the research focused exclusively on students from public universities in Ghana, limiting the generalisability of the results to private institutions. Additionally, the cross-sectional design captures only a snapshot of students’ perceptions at a single point in time, making it difficult to assess changes or trends over time. Also, while multiple institutions were included, some universities had relatively low representation, which may have skewed the findings and limited the diversity of perspectives. Furthermore, as the data was self-reported, the study may be subject to information and recall bias, which could have influenced the accuracy of participants’ responses.

### Recommendation

To enhance the clinical learning environment, academic institutions and clinical sites require coordinated efforts to address the issues of overcrowding, frequent equipment breakdowns and insufficient infrastructure with the help of policymakers. Additionally, effective communication and collaboration between universities and clinical sites will help align expectations and improve the degree of integration between theoretical knowledge and practical training. Structural orientation and mentorship programs for clinical educators can improve motivation and ensure consistency in teaching practices. It is also recommended that institutions invest in simulation laboratories to help bridge the theory and practice gap.

## Conclusion

This study highlights the perceptions of medical imaging students in Ghana regarding their clinical placements, emphasising positive experiences with supervision, learning integration, and equity. However, challenges such as overcrowding, limited supervision, and resource constraints hinder optimal learning. Addressing these issues through improved supervisor training, better coordination between academic and clinical sites, and investments in infrastructure is crucial to creating a supportive and equitable environment that prepares students for professional practice.

## Data Availability

Data required for this study may be made available by the authors upon reasonable request.
